# Pharmacist-Led Programs to Increase Statin Prescribing: A Narrative Review of the Literature

**DOI:** 10.3390/pharmacy10010013

**Published:** 2022-01-07

**Authors:** Mary Elkomos, Raha Jahromi, Michael S. Kelly

**Affiliations:** 1 Chapman University School of Pharmacy, Irvine, CA 92618, USA; elkomos@chapman.edu (M.E.); jahromi@chapman.edu (R.J.); 2 Department of Pharmacy Practice, Chapman University School of Pharmacy, Irvine, CA 92618, USA

**Keywords:** pharmacist provider, statins, statin-use measures, value-based outcomes, population health

## Abstract

Statins are lipid-lowing medications shown to reduce cardiovascular events and are recommended for specific patient populations at elevated risk of atherosclerotic cardiovascular disease (ASCVD). Despite the demonstrated efficacy of statins for reducing ASCVD risk, and guidance on which populations should receive statin therapy, a substantial portion of eligible patients are not prescribed statin therapy. Pharmacists have attempted to increase the number of eligible patients receiving appropriate statin therapy through a variety of interventions and across several clinical settings. In this article, we highlight multiple studies evaluating the effectiveness of pharmacist-led interventions to improve statin use. A total of seven studies were selected for this narrative review, demonstrating the effectiveness and barriers of different statin-initiation programs delivered by pharmacists to increase statin use in eligible patients. Among the interventions assessed, a combination of provider communicating and statin prescribing through collaborative drug therapy management (CDTM) appear to the be the most useful at increasing statin use. Pharmacists can significantly improve statin use rates among eligible patients through multiple intervention types and across different clinical settings. Further studies should evaluate continued statin adherence and clinical outcomes among patients served by pharmacists.

## 1. Introduction

Cardiovascular disease (CVD) remains the leading cause of death in the world [1]. In the United States, deaths due to CVD increased by nearly 5% from 2019 to 2020 [2]. The top two contributors to CVD mortality are coronary heart disease and stroke [1]. Prevalence of CVD increases with age, affects a greater proportion of men than women, and is more common in certain race/ethnicity groups such as non-Hispanic black patients [1]. Interventions to reduce the risk of CVD are multifactorial and include non-pharmacologic lifestyle changes and medications to treat modifiable major cardiovascular risk factors such as tobacco use, hypertension, diabetes mellitus (DM), and dyslipidemia [3,4]. 

Among the medications used to treat dyslipidemia, the HMG-CoA reductase inhibitor class of medications (or statins), are recommended as first-line agents in patients with an elevated risk of atherosclerotic cardiovascular disease (ASCVD) [3]. In 2013, updated United States guidelines for reducing cholesterol and ASCVD risk identified four statin benefit groups in which treatment with a statin medication is recommended to reduce primary or secondary ASCVD events [3]. These four statin benefit groups include: (1) patients with clinical ASCVD; (2) severe hypercholesterolemia; (3) patients with DM age 40–75 years; (4) patients without DM age 40–75 years, but with an elevated 10-year ASCVD risk. Despite clearly defined populations likely to benefit from statin therapy, the proportion of eligible patients receiving guideline recommended statin therapy is sub-optimal. In a study by Tong et al., statin use patterns were assessed among 223,289 patients from 45 clinical sites within 8 U.S. states following the publication of the 2013 ACC/AHA cholesterol guidelines [5]. While statistically significant improvements in the proportion of ASCVD patients prescribed high-intensity statin in the 2-year period following the 2013 cholesterol guideline were reported, only 20.5% of patients with ASCVD were prescribed a high-intensity statin. Similarly, less than half (44.9%) of patients aged 40–75 years with DM were prescribed any intensity of statin 2 years after the 2013 cholesterol guideline publication [5]. 

How best to address this gap of appropriate statin therapy use in the recommended benefit groups remains unclear. Currently, outcome measures related to statin use are used by several healthcare organizations, including the Centers for Medicare and Medicaid Services (CMS), National Committee for Quality Assurance (NCQA), and the Pharmacy Quality Alliance (PQA) to encourage effective treatment with statins in two patient populations: (1) statin use in patients with persons with cardiovascular disease (SPC); (2) statin use in persons with DM (SUPD) [6,7,8]. Performance on these and other healthcare measures can be used to evaluate health plans and may be linked with financial incentives [9]. As more health systems and healthcare payers transition from fee-for-service to a value-based payment model, financial reimbursement will be tied to health systems’ success in achieving clinical outcome metrics and best practice utilization [9]. Clinical pharmacist’s interventions have been associated with improved clinical outcomes related to cardiovascular risk factors, adherence to chronic medication therapy, and improved population health metrics [10,11,12]. Given their success with improving medication use and disease control, health systems are utilizing clinical pharmacists to improve statin use measures in diverse ways.

In this narrative review, we highlight multiple pharmacist-led interventions aimed at improving statin use among different patient populations and across various clinical settings. The goal of this article is to identify published articles assessing pharmacist-led interventions to increase the use of statins and discuss the potential advantages and disadvantages of each intervention.

## 2. Materials and Methods

### Literature Review

For this narrative review, our intent was not to conduct a comprehensive literature review of all published data evaluating pharmacist-led initiatives to improve statin use as would be utilized by a systematic review. Instead, we sought to provide an overview of published literature and highlight select studies showcasing various pharmacist-led statin initiation programs and to discuss the strengths and weaknesses of each.

A literature search was conducted in May 2021 across several databases (PubMed, CINAHL Plus, ScienceDirect). Keywords related to, “statin”, “statins”, “statin-initiation”, “pharmacist”, or “pharmacists” were used to identify articles which evaluated change in statin use among a population of statin non-users as a result of a pharmacist-led intervention. Since the statin use measures (SUPD, SPC) are most commonly used by healthcare organizations (Table 1), we included studies evaluating outcomes in patients aged 40–75 years with DM, or patients with cardiovascular disease, consistent with guideline recommendations. Articles reporting primary outcomes such as change in lipid parameters or statin adherence were not included. Citations from included articles were also evaluated for inclusion. 

Potential articles were independently reviewed by each author for inclusion based on the previously mentioned criteria. A total of 7 articles were selected and agreed upon by all authors after group discussion. These articles were selected based on their pharmacist-led statin initiation interventions across various clinical settings.

## 3. Results

Descriptions of the seven trials are presented below and in Table 2. Discussion of articles are presented by practice setting: post-hospital discharge; community pharmacy and outpatient primary care settings.

### 3.1. Post-Hospital Discharge

One of the earliest trials assessing the effect of pharmacist intervention on statin utilization was by Hillman et al. [13] who evaluated a post-hospital pharmacist-delivered intervention which prompted physicians to optimize statins therapies in patients discharged from a university-affiliated teaching hospital. Patients admitted to the coronary care unit and diagnosed with coronary heart disease were assigned to control (n = 303) or intervention (n = 309) groups based on admission date. Patients in the control group received no pharmacist intervention after hospital discharge. In the intervention group, pharmacists contacted patients’ provider via mailed letters and phone calls with specific recommendations for statin initiation or optimization based on patients’ lipid values at 2, 8-, 12-, 24-, and 52-weeks post hospital discharge. 

Baseline characteristics of the study groups were similar. At hospital discharge, slightly more patients in the control group were treated with a statin compared to the intervention group (39% vs. 35%). Both groups had similar rates of prior myocardial infarction (16%). After 104 weeks, 43% of patients were on statin therapy in the control group versus 72% of patients in the intervention group. A greater proportion of the intervention group had low-density lipoprotein cholesterol (LDL-C) values at goal by week 104 compared to the control group (55% vs. 18%; *p* < 0.05). Additionally, patients in the intervention group had significantly lower clinical events (recurrent myocardial infarction, hospitalization for myocardial ischemia, coronary revascularization, percutaneous coronary intervention, and cardiovascular mortality) when compared with the control group. In patients hospitalized for coronary heart disease, a pharmacist-led intervention after hospital discharge significantly improved statin-use rates and was associated with reduced cardiovascular events over a 2-year period.

### 3.2. Community Pharmacy Setting

A pilot study conducted within 4 community outpatient pharmacies in Idaho evaluated the effectiveness and patient perspectives of a pharmacist statin prescribing service [14]. The service was aimed to improve statin use among patients aged 40–75 years with a diagnosis of type 2 DM, but without an active statin prescription. Patients meeting criteria were identified using the electronic quality improvement platform for plans and pharmacies (EQuIPP) database. Eligible patients were contacted by pharmacists via phone call (up to 3 attempts) or by placing a note for pharmacist consultation in the patient’s filled prescriptions. Patients who agreed to the statin service were scheduled for an in-person appointment at the pharmacy to complete a baseline liver function test before prescribing a statin. Following a normal liver function result, pharmacists prescribed and dispensed statin therapy and notified the patient’s primary care provider (PCP). A “nominal” fee was required of patients who met with the pharmacist and were prescribed statin to cover the cost of the liver function test and the pharmacist’s time.

A total of 64 eligible patients were identified from the EQuIPP database, of which 33% had a statin contraindication, 27% could not be contacted, and 19% of patients refused. Reasons for patient refusal were assessed and included the out-of-pocket cost, preference for prescriptions to come from their primary care provider, and not understanding the purpose of a statin. Only 6 patients (9.4%) agreed to the pharmacist service and a total of 4 patients (6.2%) were prescribed a statin; 2 patients (50%) received a statin prescription from pharmacists, while the other 2 patients requested a statin prescription from their PCP due to delayed liver function results. Although the effectiveness of this pharmacist-led statin prescribing service was low, it may serve as a template for other community pharmacies looking to increase statin use in the eligible DM population. The fee for the liver function testing and pharmacist service may be a barrier for engaging patients and thus alternative payment options for clinical pharmacist services are warranted.

Renner et al. [15] assessed the effectiveness of a pharmacist-led intervention in a community pharmacy setting to initiate statins in patients with DM aged 40–75 years, without an active statin prescription. Patients were randomized to receive the pharmacist intervention or no intervention (control). In the intervention group pharmacists contacted patients’ PCP via phone and up to two follow-up fax messages requesting a statin prescription. Reasons for provider refusal were also documented. 

The control group had more males (51.7% vs. 47%) when compared to the intervention group. Both groups had a mean age of 66 years. After 3 months, a greater proportion of patients in the intervention group were initiated on statins versus patients in the control group (20.8% vs. 8.5%; *p* < 0.001). Approximately 70% of statin prescriptions were received after the initial phone or first fax message. The number of statins dispensed was significantly higher with the pharmacist intervention compared to control (15.4% versus 7.5%; *p* = 0.015). The top reasons for provider refusal of statin initiation were prior statin intolerance (23.3%), and PCP preferred to see the patient before initiating a statin (20.8%). This study shows that a somewhat straightforward pharmacist intervention of messaging providers led to greater statin initiation and dispensed prescription rates among patients with DM. Pharmacist access to such data is essential in determining medication gaps in therapy in order to intervene.

### 3.3. Outpatient Primary Care Clinics

In an outpatient primary care clinic, Vincent et al. [16] assessed the impact of a pharmacist statin-initiation program in patients with DM. The study assessed the percentage of patients prescribed statin therapy before and 3 months after the statin program was initiated. The pharmacist statin-initiation program utilized an electronic medical record (EMR) report to identify patients aged 40 to 75 years of age with DM, and an upcoming PCP appointment within the next 3 months. This report also identified which patients had an active statin prescription listed in the EMR medication list. The pharmacist intervention to improve statin utilization included recommending statins to providers through face-to-face communication, or EMR messaging. If the physician accepted the pharmacist’s recommendation, statin initiation could be facilitated by the PCP or pharmacist. 

At baseline, a total of 454 patients with DM were identified with the EMR report; most patients (75.6%) had an active prescription for statin therapy. Baseline characteristics of the total study population included a mean age of 58 years, nearly 60% female, 76.4% African American, and 32% had ASCVD. After 3 months, the percentage of patients with an active statin prescription increased to 82.6% (*p* < 0.0001 compared to baseline). Among the 111 patients (24.4%) without an active statin prescription at baseline, pharmacists made a total of 61 recommendations, 90.2% were accepted by physicians, but only 32 recommendations (52.5%) to initiate statin therapy were implemented, thus only 28.8% of patients not receiving statin therapy at baseline-initiated treatment during the 3-month study period. Notably, 23 out of 61 pharmacist recommendations (37.7%) were deferred by the PCP until a future appointment. In total, recommending interventions to be carried out by primary care physicians during outpatient appointments may not be an ideal way to increase statin initiation among recommended populations. 

Haby et al. [17] investigated a population health intervention delivered by clinical pharmacists across 10 primary care clinics in Washington to increase appropriate statin use among patients aged 21–75 years with a diagnosis of ASCVD. A report of patients with ASCVD was generated from the EMR. Identified patients were then screened for eligibility by pharmacy students and residents, followed by pharmacist review to ultimately determine statin eligibility. Patients with documented statin intolerance or an erroneous ASCVD diagnosis were excluded, and their EMR was updated accordingly by pharmacists. Remaining eligible patients were stratified to two pharmacist intervention types based on the date of future PCP appointment. For patients with an appointment within the next 2 months, pharmacists coordinated with their PCP to discuss statin initiation at that visit. Patients without an appointment in the next 2 months were directly contacted by pharmacists to verify past medical history and discuss statin treatment. Pharmacists could then directly prescribe appropriate statin therapy through a health system protocol. 

A total of 510 patients were screened for statin appropriateness; however, pharmacist intervention was attempted for 307 patients (60.2%). After revising the EMR of 62 patients, 245 patients remained eligible for pharmacist intervention. Most patients (76%) were contacted through direct patient outreach. Overall, 98 patients accepted pharmacists’ recommendations for statin initiation (40%). Evaluating statin initiation rates by intervention found the highest success with PCP clinic coordination (53.3%), compared to direct outreach (36%). Among patients who did not initiate statin therapy, 32% could not be reached and 27% declined. From this population health study, it appears that direct patient outreach and independent pharmacist statin prescribing are effective ways to increase appropriate statin use in high-risk patients, although less successful than coordinated efforts with patients’ PCP.

Two pharmacist-led interventions (upcoming appointment and dual-strategy) to initiate statins in patients with type 2 DM age 40 to 75 years was assessed by Troska et al. [18]. The upcoming appointment intervention utilized pharmacists embedded within the clinic to identify eligible patients with an upcoming PCP visit within the next 7 days. The electronic health record specifically identified patients aged 40–75, diagnosed with type 2 DM, currently not receiving statin therapy and with no diagnosis of ASCVD. Pharmacists documented their recommendation for statin therapy in an EMR and forwarded the recommendation to the patient’s PCP, in addition to adding a note about statin eligibility to the PCP’s schedule. The dual-strategy intervention method utilized centrally located pharmacists to recommend statin therapy using the upcoming appointment approach, or via a second intervention (prospective panel approach) consisting of sending a list of eligible patients without upcoming appointments to PCPs. Providers reviewed this list and indicated which patients could be contacted and imitated on statin therapy by the pharmacist.

A random sample of 400 patients (n = 200 from each intervention strategy) were chosen for analysis of the primary endpoint. Patients in the dual-strategy cohort were older (61.2 vs. 58.1 years) and had a higher 10-year ASCVD risk (19.4% vs. 15.6%) compared to the upcoming appointment cohort. Most patients (55.5%) in the dual-strategy cohort were recommended statin therapy by the upcoming appointment approach. Over an 8-month study period, statin initiation was higher using the upcoming appointment method compared to the dual strategy method (46% vs. 36%; *p* = 0.042). Compared to the prospective panel approach, the upcoming appointment approach resulted in significantly greater statin prescribing rates (31.5% vs. 42.9%; *p* = 0.049). Among this cohort of 400 patients, statin therapy was not initiated in 59.5%, most commonly because statin therapy was not discussed at the PCP visit, or the patient declined. This study is the first to assess the effectiveness of two different pharmacist statin initiation interventions, which supports previous findings that informing physicians about statin eligibility prior to an upcoming patient appointment resulted in a higher percentage of statin initiation. 

A prospective trial by Anderson et al. [19] assessed the effectiveness of clinical pharmacists embedded within several patient centered medical home (PCMH) clinics to improve the statin use in persons with DM (SUPD) measure among a managed care population. A list of patients was received aged 40–75 with at least two medication fills for DM with no statin prescription. Clinical pharmacists reviewed patient eligibility and contacted patients who met the SUPD criteria. Clinical pharmacists were able to independently prescribe statin therapy to patients previously referred for pharmacist management using an existing collaborative drug therapy management (CDTM) protocol. For patients not already referred for pharmacist disease management, statin therapy could be prescribed by pharmacists but required a provider co-signature.

At the baseline, 1022 patients were identified as eligible for the SUPD criteria, but a majority (79.5%) were excluded due to already receiving statins. A remaining 339 patients were identified during the 11-month study period; mean age was 63 years; mean glycosylated hemoglobin A1c was 8.0%; and mean ASCVD risk score was 21.7%. After chart review and contacting 326 patients, only 275 (84.4%) were considered eligible to add statin therapy. Among these patients, 41.8% were prescribed statin therapy. High-intensity statin was prescribed in 60.9% of patients. Among patients who were not prescribed statin therapy, the top reasons were patient refusal (23.3%), patients reportedly out of the country (17.1%), and unable to reach patients after several phone calls (14.9%). Only six providers refused to prescribe a statin, usually due to wanting to see the patient in person to discuss. A CDTM protocol successfully allowed pharmacists to initiate statin therapy and improve the SUPD measure within a PCMH; however, a large proportion of patients identified as not meeting the measure were in fact prescribed statin therapy. Significant time and effort are needed to refine lists generated by health plans to identify patients not receiving recommended treatment.

## 4. Discussion

From our review, several types of pharmacist-led interventions significantly increase statin prescriptions across a variety of clinical settings. The most common types of interventions studied include physician prompting by the pharmacist via messaging or direct-patient contact with pharmacist prescribing. Simply notifying providers that a patient with an upcoming appointment is eligible for statin therapy was somewhat successful in initiating statin therapy, as described by Vincent et al. [16]. Two trials evaluated two different types of pharmacy interventions, direct-patient outreach versus coordinating with providers about statin eligibility prior to an upcoming patient appointment [17,18] each reporting a statistically greater proportion of patients prescribed statin therapy with the provider coordination intervention vs. the direct-patient outreach intervention. However, Anderson et al. [19] reported somewhat similar success with a direct-patient outreach intervention which allowed pharmacists to prescribe statins in consenting patients via a CDTM protocol. In each of these studies, pharmacists were to some degree already implemented in these clinical sites, suggesting a positive provider-pharmacist relationship may have already been formed. Comparatively, two trials from community pharmacy settings [14,15] reported a positive impact on improving statin prescriptions in eligible patients, but had lower success rates than other trials, even with independent statin prescribing privileges [14]. This finding could be a result of no established pharmacist-provider relationship. In a trial by Renner et al., a greater proportion of patients were prescribed statin therapy as a result of pharmacist intervention versus no-intervention, yet only approximately 1 out of 5 eligible patients was prescribed a statin by their provider [15]. In a trial by Spann et al., pharmacists could independently prescribe statin therapy to consenting patients, yet only 2 patients received a statin prescription from a pharmacist [14]. By far the most successful intervention was seen in the study by Hileman et al., which showed that pharmacist messaging to providers resulted in significant improvements in patients prescribed statin therapy, achieving LDL-C goals, and seemed to reduce several cardiovascular outcomes over a 2-year follow-up period [13]. Potential reasons for such high success reported in this trial may be due to the academic hospital setting, where a more collaborative and patient-centered culture exists, the longer study period of 2 years, or that the study population was at high risk given that they were admitted to the coronary care unit. Subsequent cost-analysis of this trial evaluated total cost of care for the intervention and control groups [20]. The total cost of care included pharmacist’s salary, materials for messaging providers, costs associated with clinic and laboratory tests, as well as the costs of subsequent cardiovascular events. Across the 2-year study period, total costs per patient were lower in the intervention group compared to the control group (USD $3474 vs. USD $5747), resulting in an estimated savings of $1394 per patient. This cost savings was driven by reduced costs associated with subsequent cardiovascular events.

The first step in implementing any program aimed to increase statin prescriptions is obtaining reliable data on eligible patients. Several studies demonstrated that among the reportedly eligible patients, a significant number were excluded due to already receiving statin therapy or because of an exclusion (i.e., statin intolerance). In the study by Haby et al., among the 510 patients initially identified as not meeting the SUPD metric, 16.5% of patients screened were excluded as they were deemed not appropriate due to inclusion or exclusion metric reporting error, recent discontinuation of statin by patient or provider, or the patient had not seen their provider in over 1-year [17]. In the trial by Anderson et al., 79% of patients initially identified as not meeting the SUPD measure were excluded because they were already prescribed a statin [19]. Utilizing other non-clinical staff such as pharmacy technicians or pharmacy students may be an effective way to review patient charts for eligibility to maximize clinical pharmacists’ time delivering patient care.

In trials where pharmacists directly outreached to patients about statin eligibility, a large proportion of patients declined [14,18,19]. Reported reasons for patients declining included that they were not aware of the benefits or felt they did not need the statin [14]. This finding suggests that pharmacists who outreach to patients will need to be able to adequately describe the benefits and risks of statin therapy. A 2019 systematic review of interventions to increase statin prescribing in primary prevention populations found that interventions aimed at educating patients were more successful at increasing statin-prescription rates compared to physician education and decision-support interventions [21]. Several organizations provide patient-friendly material on statin treatment which may be useful in describing the real vs. perceived benefits and adverse effects [22,23,24]. 

Lastly, once statins have been prescribed there exists additional roles for pharmacists to ensure patients continue to take them. In addition to the SUPD and SPC measure, several agencies also evaluate the portion of days covered (PDC) statin measure as well. Pharmacists have demonstrated significant abilities to improve adherence to statin therapy through a variety of methods [25]. A randomized cluster trial evaluated the effect of a multicomponent, individualized behavioral intervention delivered remotely by pharmacists on medication adherence versus no intervention in patients prescribed chronic medication for hypertension, DM, or dyslipidemia within a large medical group [26]. Over a 12-month period, medication adherence was 4.7% higher in the intervention group compared to control, despite no significant improvement in disease control in 1 or more conditions but was associated with a 38% reduction in emergency department visits. As such, pharmacist interventions to increase statin prescribing should be tied to individualized interventions to maintain statin adherence for optimal patient outcomes.

Limitations of our review include potential selection bias as authors were responsible for selecting included articles for this narrative review, potentially excluding other relevant work. Additionally, compared to systematic reviews narrative reviews typically do not assess the quality of included studies [27]. While it may be possible that other relevant articles were overlooked, we believe our literature search and selection process are sufficient to provide a broad overview on the topic pharmacist interventions to improve statin use, as is the intent of narrative reviews [27]. Secondly, all but one article assessed a primary prevention population with diabetes aged 40–75 years, thus generalizability to other statin benefit groups may not be applicable. Despite current cholesterol guidelines recommending statin therapy for patients aged 40–75 years with DM [28], some patients are not prescribed statin therapy for primary prevention as they or their prescriber believe their cholesterol values to be controlled [15,17,19]. Given that elevated cholesterol is associated with increased ASCVD risk, it is not the only factor to consider when determining whether a patient with DM should receive statin therapy for primary prevention. A recent meta-analysis assessed the risk of ASCVD events among patients with DM, but without cardiovascular disease, and LDL-C values of 100 to 159 mg/dL [29]. From the eleven trials included, statin therapy was associated with a lower risk of composite cardiovascular events (RR = 0.71; 95% CI, 0.62–0.82) as well as coronary revascularizations and cardiovascular hospitalizations [29]. This adds to growing data suggesting statins are effective in reducing ASCVD events in primary prevention patients with mildly elevated LDL-C.

## 5. Conclusions

Multiple pharmacist-led interventions have led to increased statin use and are likely to improve statin-related outcome metrics; however, a substantial number of patients do not receive guideline recommended statin therapy. The most effective interventions to increase statin use appear to include pharmacist prompting of providers in settings where an established relationship exists between provider and pharmacist. 

Barriers to increasing statin use in high-risk patient populations include erroneous patient eligibility data and patient’s declining statin therapy. Further studies assessing interventions to resolve patient and provider barriers to increasing statin use in high-risk groups is needed to reduce the number of patients without statin treatment and lower the global burden of CVD. Health systems looking to improve metrics related to statin use should select an intervention that best aligns with their patient population, clinical pharmacy workforce, and health system structure. Assessing avoided ASCVD events in patients treated with statin therapy would be an important outcome of future studies.

## Figures and Tables

**Table 1:** Inclusion and exclusion criteria for statin use measures by organization.

Statin Use in Persons with Diabetes (SUPD)		
	Inclusion criteria	Exclusion criteria ^a^
CMS [6]	Age 40–75 years old who were dispensed at least two diabetes medication fills	Hospice enrollment ESRD
HEDIS [8]	Age 40–75 years, diagnosed with diabetes or have at least 2 refills of a diabetes medication	Cardiovascular disease ESRD Cirrhosis SAMS Pregnancy Palliative care
PQA [7]	Age 40 to 75 years who were dispensed a medication for diabetes	Hospice enrollment ESRD Liver disease SAMS Pregnancy Pre-diabetes PCOS
**Statin Therapy for Patients with Cardiovascular Disease (SPC)**		
CMS [6]	Males aged 21–75 years; females 40–75 years with ASCVD and were dispensed at least one high or moderate-intensity statin medication	ESRD Cirrhosis SAMS Pregnancy
HEDIS [8]	Males aged 21–75 years; females 40–75 years of age who have clinical ASCVD and who received statin therapy	Hospice enrollment

**^a^** Select exclusion criteria listed; ASCVD = Atherosclerotic Cardiovascular Disease; CMS = Centers for Medicare and Medicaid Services; ESRD = End-Stage Renal Disease; HEDIS = Healthcare Effectiveness Data and Information Set; PCOS = Polycystic Ovarian Syndrome; PQA = Pharmacy Quality Alliance; SAMS = Myalgia, myositis, myopathy, or rhabdomyolysis.

**Table 2:** Selected articles describing pharmacist-led statin initiation outcomes.

Study	Clinical Setting	Study Population	Pharmacist Intervention to Improve Statin Use	Study Duration	Results
Hilleman et al. [13]	Post-hospital discharge	Patients discharged from hospital following admission to coronary care unit for CHD Control (n = 303) Intervention (n = 309)	Intervention group—Phone or mailed communication to patient’s PCP regarding statin therapy Control group—no pharmacist intervention	104 weeks	Proportion of patients prescribed statin therapy at week 104 (intervention vs. control): 72% vs. 43%; *p* < 0.05 MACE events at week 104 (intervention vs. control): Hospitalization for MI (15% vs. 23%) Coronary revascularization (12% vs. 21%) Cardiovascular mortality (9% vs. 12%) *p* < 0.05 for all MACE outcomes
Spann et al. [14]	4 community pharmacies in Idaho	Patients aged 40–75 years with T2DM, without active statin prescription	Patient outreach and independent pharmacist statin prescribing	3 months	64 eligible patients: 4 patients (6.25%) initiated statins 2 patients (3.12%) prescribed statin from Pharmacists
Renner et al. [15]	Community pharmacy	Patients aged 40–75 years with DM, without active statin prescription Control (n = 199) Intervention (n = 221)	Intervention group—Phone and fax messages to patient’s PCP to initiate statin Control—no pharmacist intervention	3 months	Proportion of patients prescribed statin therapy (Intervention vs. control): 20.8% vs. 8.5%; *p* < 0.001 Dispensed statin prescriptions (intervention vs. control) 15.4% vs. 7.5%; *p* = 0.015
Vincent et al. [16]	Primary care clinic with embedded clinical pharmacy services	Patients aged 40–75 years with DM, without active statin prescription, and upcoming PCP appointment	Pharmacist notifies PCP of patient eligibility prior to appointment	3 months	111 eligible patients: 28.8% of patients prescribed statin
Haby et al. [17]	10 primary care clinics with embedded clinical pharmacy services	Patients aged 21–75 years with diagnosis of ASCVD not receiving moderate or high-intensity statin (n = 307)	Direct patient outreach and pharmacist statin prescribing Coordinating with PCP about statin use prior to upcoming patient visit	3 months	245 eligible patients: 40% agreed to pharmacist-recommended statin therapy Percent of patients agreeing to statin by intervention type: Coordinating with PCP = 53.3% Direct patient outreach = 36.0%
Troska et al. [18]	Embedded and centrally located clinical pharmacists	Patients aged 40–75 years with DM, without active statin prescription Single strategy (n = 200) Dual strategy (n = 200)	Single strategy: Pharmacist notifies PCP of patient with upcoming appointment and statin eligibility Dual strategy: Either the single strategy above or pharmacist sends list of eligible patients to provider to receive approval to contact patient and initiate statin if patient agrees	8 months	Proportion of patients prescribed statins (single strategy vs. dual strategy) 46% vs. 36%; *p* = 0.042 Proportion of patients prescribed statins (upcoming appointment vs. list of eligible patients) 42.9% vs. 31.5%; *p* = 0.049
Anderson et al. [19]	Patient- centered medical home (PCMH) clinics with embedded clinical pharmacy services	Patients aged 40–75 years with DM, without active statin prescription	Patient outreach and pharmacist statin prescribing through CDTM or provider co-signature	11 months	275 eligible patients: 41.8% of patients prescribed statin

ASCVD = Atherosclerotic Cardiovascular Disease; CDTM = Collaborative Drug Therapy Management; CHD = Coronary Heart Disease; DM = Diabetes Mellitus; MACE = Major Adverse Cardiovascular Events; PCP = Primary Care Provider; T2DM = Type 2 Diabetes Mellitus.

## References

[1] Virani S.S., Alonso A., Aparicio H.J., Benjamin E.J., Bittencourt M.S., Callaway C.W., Carson A.P., Chamberlain A.M., Cheng S., Delling F.N. (2021). Heart Disease and Stroke Statistics—2021 Update: A Report From the American Heart Association. Circulation.

[2] Ahmad F.B., Anderson R.N. (2021). The Leading Causes of Death in the US for 2020. JAMA.

[3] Stone N., Robinson J.G., Lichtenstein A.H., Merz C.N.B., Blum C.B., Eckel R.H., Goldberg A.C., Gordon D., Levy D., Lloyd-Jones D. (2013). 2013 ACC/AHA Guideline on the Treatment of Blood Cholesterol to Reduce Atherosclerotic Cardiovascular Risk in Adults. Circulation.

[4] Arnett D.K., Blumenthal R.S., Albert M.A., Buroker A.B., Goldberger Z.D., Hahn E.J., Himmelfarb C.D., Khera A., Lloyd-Jones D., McEvoy J.W. (2019). 2019 ACC/AHA Guideline on the primary prevention of cardiovascular disease: A report of the american college of cardiology/American heart association task force on clinical practice guidelines. Circulation.

[5] Tong S.T., Sabo R.T., Hochheimer C.J., Brooks E.M., Jiang V., Huffstetler A.N., Kashiri P.L., Krist A.H. (2021). Uptake of Statin Guidelines to Prevent and Treat Cardiovascular Disease. J. Am. Board Fam. Med..

[6] The Centers for Medicare and Medicaid Services (CMS) Fact Sheet-2022 Part C and D Star Ratings. http://go.cms.gov/partcanddstarratings.

[7] PQA Measures Overview. https://www.pqaalliance.org/measures-overview.

[8] Statin Therapy for Patients With Cardiovascular Disease and Diabetes—NCQA. https://www.ncqa.org/hedis/measures/statin-therapy-for-patients-with-cardiovascular-disease-and-diabetes/.

[9] What Is Pay for Performance in Healthcare?. https://catalyst.nejm.org/doi/full/10.1056/CAT.18.0245.

[10] Hartkopf K.J., Heimerl K.M., McGowan K.M., Arndt B.G. (2020). Expansion and Evaluation of Pharmacist Services in Primary Care. Pharmacy.

[11] AlShehri A.A., Jalal Z., Cheema E., Haque M.S., Jenkins D., Yahyouche A. (2019). Impact of the pharmacist-led intervention on the control of medical cardiovascular risk factors for the primary prevention of cardiovascular disease in general practice: A systematic review and meta-analysis of randomised controlled trials. Br. J. Clin. Pharmacol..

[12] Dixon D.L., Khaddage S., Bhagat S., Koenig R.A., Salgado T.M., Baker W.L. (2020). Effect of pharmacist interventions on reducing low-density lipoprotein cholesterol (LDL-C) levels: A systematic review and meta-analysis. J. Clin. Lipidol..

[13] Hilleman D.E., Monaghan M.S., Ashby C.L., Mashni J.E., Woolley K., Amato C.M. (2001). Physician-prompting statin therapy intervention improves outcomes in patients with coronary heart disease. Pharmacother. J. Hum. Pharmacol. Drug Ther..

[14] Spann N., Hamper J., Griffith R., Cleveland K., Flynn T., Jindrich K. (2020). Independent pharmacist prescribing of statins for patients with type 2 diabetes: An analysis of enhanced pharmacist prescriptive authority in Idaho. J. Am. Pharm. Assoc..

[15] Renner H.M., Hollar A., Stolpe S.F., Marciniak M.W. (2017). Pharmacist-to-prescriber intervention to close therapeutic gaps for statin use in patients with diabetes: A randomized controlled trial. J. Am. Pharm. Assoc..

[16] Vincent R., Kim J., Ahmed T., Patel V. (2019). Pharmacist Statin Prescribing Initiative in Diabetic Patients at an Internal Medicine Resident Clinic. J. Pharm. Pract..

[17] Haby H.E., Alm R.A., Corona A.R., Hall A.C. (2020). Population health model for pharmacist assessment and independent prescribing of statins in an ambulatory care setting. J. Am. Pharm. Assoc..

[18] Troksa K.A., Billups S.J., Claus L.W., Griend J.P.V., Saseen J.J. (2020). Effectiveness of a pharmacist-led population health approach to implementing statin therapy in primary prevention patients with type 2 diabetes mellitus. J. Am. Coll. Clin. Pharm..

[19] Anderson S., Marrs J.C., Chachas C.R., Cichon B.S., Cizmic A.D., Calderon B.B., Vlasimsky T.B. (2020). Evaluation of a Pharmacist-Led Intervention to Improve Statin Use in Persons with Diabetes. J. Manag. Care Spéc. Pharm..

[20] Hilleman D.E., Faulkner M.A., Monaghan M.S. (2004). Cost of a Pharmacist-Directed Intervention to Increase Treatment of Hypercholesterolemia. Pharmacother. J. Hum. Pharmacol. Drug Ther..

[21] Sparrow R.T., Khan A.M., Ferreira-Legere L.E., Ko D., Jackevicius C.A., Goodman S.G., Anderson T.J., Stacey D., Tiszovszky I., Farkouh M.E. (2019). Effectiveness of Interventions Aimed at Increasing Statin-Prescribing Rates in Primary Cardiovascular Disease Prevention. JAMA Cardiol..

[22] Patient Tear Sheets | National Lipid Association Online. https://www.lipid.org/patient-tear-sheets.

[23] Cholesterol Patient Education Handouts|cdc.gov. https://www.cdc.gov/cholesterol/materials_for_patients.htm.

[24] Statin Choice Decision AID Site. https://statindecisionaid.mayoclinic.org/.

[25] Elnaem M.H., Rosley N.F.F., Alhifany A.A., Elrggal M.E., Cheema E. (2020). Impact of Pharmacist-Led Interventions on Medication Adherence and Clinical Outcomes in Patients with Hypertension and Hyperlipidemia: A Scoping Review of Published Literature. J. Multidiscip. Health.

[26] Choudhry N.K., Isaac T., Lauffenburger J., Gopalakrishnan C., Lee M., Vachon A., Iliadis T.L., Hollands W., Elman S., Kraft J.M. (2018). Effect of a Remotely Delivered Tailored Multicomponent Approach to Enhance Medication Taking for Patients with Hyperlipidemia, Hypertension, and Diabetes. JAMA Intern. Med..

[27] Narrative Review-Reviews: From Systematic to Narrative-Research Guides at University of Alabama-Birmingham. https://guides.library.uab.edu/c.php?g=63689&p=409774.

[28] American Diabetes Association 10 (2020). Cardiovascular Disease and Risk Management: Standards of Medical Care in Diabetes—2021. Diabetes Care.

[29] Singh B.M., Lamichhane H.K., Srivatsa S.S., Adhikari P., Kshetri B.J., Khatiwada S., Shrestha D.B. (2020). Role of Statins in the Primary Prevention of Atherosclerotic Cardiovascular Disease and Mortality in the Population with Mean Cholesterol in the Near-Optimal to Borderline High Range: A Systematic Review and Meta-Analysis. Adv. Prev. Med..

